# A health economic pilot study comparing two diabetic retinopathy screening strategies

**DOI:** 10.1038/s41598-024-66405-2

**Published:** 2024-07-06

**Authors:** Ellen Steffenssen Sauesund, Silvia N. W. Hertzberg, Øystein Kalsnes Jørstad, Morten Carstens Moe, Maja Gran Erke, Dag Sigurd Fosmark, Goran Petrovski

**Affiliations:** 1 Department of Ophthalmology, Oslo University Hospital, 0450 Oslo, Norway; 2 Center for Eye Research and Innovative Diagnostics, Department of Ophthalmology, Institute of Clinical Medicine, Faculty of Medicine, University of Oslo, 0450 Oslo, Norway; 3 University of Split, School of Medicine, 21000 Split, Croatia; 4 UKLONetwork, University St. Kliment Ohridski-Bitola, 7000 Bitola, North Macedonia

**Keywords:** Diabetic retinopathy, Diabetic maculopathy, Diabetic macular edema, OCT, Screening, Cost-savings, Epidemiology, Outcomes research

## Abstract

To compare two screening strategies for diabetic retinopathy (DR), and to determine the health-economic impact of including optical coherence tomography (OCT) in a regular DR screening. This cross-sectional study included a cohort of patients (≥ 18 years) with type 1 or 2 diabetes mellitus (T1D or T2D) from a pilot DR screening program at Oslo University Hospital, Norway. A combined screening strategy where OCT was performed in addition to fundus photography for all patients, was conducted on this cohort and compared to our existing sequential screening strategy. In the sequential screening strategy, OCT was performed on a separate day only if fundus photography indicated diabetic macular edema (DME). The presence of diabetic maculopathy on fundus photography and DME on OCT was determined by two medical retina specialists. Based on the prevalence rate of diabetic maculopathy and DME from the pilot, we determined the health-economic impact of the two screening strategies. The study included 180 eyes of 90 patients. Twenty-seven eyes of 18 patients had diabetic maculopathy, and of these, 7 eyes of 6 patients revealed DME on OCT. When diabetic maculopathy was absent on fundus photographs, OCT could not reveal DME. Accordingly, 18 patients (20%) with diabetic maculopathy would have needed an additional examination with OCT in the sequential screening strategy, 6 (33%) of whom would have had DME on OCT. In an extended healthcare perspective analysis, the cost of the sequential screening strategy was higher than the cost of the combined screening strategy. There was a weak association between diabetic maculopathy on fundus photography and DME on OCT. The health economic analysis suggests that including OCT as a standard test in DR screening could potentially be cost-saving.

## Introduction

The global prevalence of diabetes mellitus (DM) (age: 20–79 years) in 2021 was approximately 537 million, estimated to rise to 783 million by 2045^1,2^. Diabetic retinopathy (DR) is a common microvascular complication of DM, which stands as the primary contributor to worldwide blindness among individuals aged 50 years and above; DR-related complications are the main cause of impaired vision in patients aged 24–74 years^3,4^. Diabetic macular edema (DME) is a serious complication of DM, which can lead to severe visual impairment if left untreated^5,6^. As the prevalence of DM worldwide is increasing, the financial burden of DM complications is substantial and rising in parallel^7–9^. Screening programs play a crucial role in early detection of sight-threatening DR, enabling timely interventions that can preserve visual function and improve patient outcomes in a cost efficient manner.

Stereoscopic seven-field fundus photography has been used in DR screening and to detect DME in accordance with the Early Treatment Diabetic Retinopathy Study (ETDRS)^10^, but it is time-consuming and requires expertise. Consequently, DR screening programs have opted out of using stereoscopic photos and instead implemented 2D imaging. The latter can predict DME by checking for associated signs of retinal thickening, such as the presence of hard exudates and microaneurysms (MAs) near the fovea^11^. Still, studies have demonstrated limited specificity and sensitivity of such surrogate markers of DME^12–14^.

While fundus photography remains a valuable tool for the initial assessment of DR, its limitations in detecting subtle changes in macular morphology and early signs of DME have prompted the integration of optical coherence tomography (OCT) into diabetic eye screening protocols. OCT has revolutionized the ability to detect DME through high-resolution images of the macula in a non-invasive, objective, and reproducible way^15,16^.

Although OCT can be considered the gold standard for detecting and monitoring DME^17–20^ in contemporary ophthalmology, it is not a routine element of DR screening. Some countries like Denmark and the UK have incorporated OCT into their national DR screening programs in an alternative manner, using it only when diabetic maculopathy is suspected. Additionally, the new DR screening guidelines in Norway now recommend this approach^21–24^.

The quantitative data obtained from OCT imaging can enhance the accuracy of diagnosing DME and monitoring disease progression over time. OCT currently guides intravitreal injection treatment of DME; typically, the indication is based on a combination of DME on OCT and loss of visual acuity^25–27^. Accordingly, an important aspect of DR screening is to detect DME that needs intervention. However, if only fundus photography is available, there is a possibility of both false positive and negative findings, challenging the value of screening for DME. One solution is to perform a second screening visit with OCT when fundus photography raises suspicion of DME. This was the existing “sequential” screening strategy in our department. An alternative solution is a “combined” screening strategy that always includes OCT in addition to fundus photography, regardless of suspicion of DME.

Despite the clinical utility of OCT in detecting and monitoring DME, its integration into routine DR screening programs has been limited by cost considerations^28^. The economic implications of incorporating OCT alongside fundus photography in DR screening protocols have been a subject of interest, while understanding the health-economic impact of combining the two modalities in DR screening is essential for optimizing resource allocation and improving the efficiency of diabetic eye care services.

In the present pilot study, we identified the prevalence of diabetic maculopathy detected on 2D fundus photography and compared it to DME detected on OCT. We also compared this combined screening strategy with the existing sequential screening strategy in our department and assessed the cost-effectiveness of routinely combining fundus photography and OCT in DR screening.

## Patients and methods

The study included a cohort of patients (≥ 18 years) with type 1 or 2 DM (T1D or T2D) from a pilot DR screening program at Oslo University Hospital (OUH), Norway, conducted during the period from December, 2019 to January, 2021.

The patients were mainly referred by general practitioners (GPs), who had received information about the pilot (12 patients were referred from another affiliated healthcare institution). The GPs were invited to refer patients without known treatment-dependent DR who were not already followed by an ophthalmologist. A total of 90 patients (180 eyes) were enrolled, of whom 12 with T1D and 78 with T2D which approximately reflects the estimated prevalence of T1D and T2D in the Norwegian population^22^. We obtained written informed consent from all patients who were included from December 2019 to January 2021. The Regional Committee for Medical and Health Research Ethics concluded that the project was outside the remit of the Norwegian Health Research Act (reference: 28857). The Institutional Data Protection Officer at OUH approved the study (reference: 20/00571), which was in accordance with the relevant guidelines and regulations.

All patients were examined at the Department of Ophthalmology, OUH. Colour fundus photography was performed using a CLARUS™ 700 camera, Zeiss (Carl Zeiss Meditec AG, Jena, Germany). Fovea- and optic-disc-centered photos were obtained, both with 133°-field images. Diabetic maculopathy based on fundus photography was classified as follows: no maculopathy (0), presence of MA within 1 disc diameter (DD) of the foveola (1), and hard exudate(s) within 1 DD of the foveola (2).

OCT macula radial scans of 6 × 6 mm were obtained using NIDEK RS-3000 Advance OCT Retina Scan (NIDEK CO., LTD, Gamagori, Japan). In this study the presence of intraretinal cysts and/or subretinal fluid in the central area of the macula, within one disc diameter from the foveal center, was determined qualitatively in our study by two experienced ophthalmologists (ESS, DF).

In the pilot DR screening we utilized a combined screening strategy where OCT was performed in addition to fundus photography on all 90 patients included. The existing DR screening strategy in our department involved performing OCT only when fundus photography showed diabetic maculopathy. Subsequently, patients were required to return for OCT on another day to evaluate the presence of DME. The prevalence of diabetic maculopathy identified through fundus photography and the prevalence of DME detected on OCT in the pilot screening were determined. These prevalence rates, along with the DME/ diabetic maculopathy ratio, were then used in the cost analyses for both screening strategies.

We conducted a cost analysis of DR screening using fundus photography and OCT combined on the same day (combined screening strategy) or different days (existing sequential screening strategy). For sequential days, all fundus photography costs were included, whereas OCT costs were based on the presence of diabetic maculopathy. We also estimated the total cost of true positives for the presence of DME. These analyses involved varying key parameters such as prevalence rates and imaging costs to assess the stability of our cost estimates under different scenarios.

Diagnosis-Related Group (DRG) is a system that categorizes patients into different groups, considering factors such as diagnosis and treatment. The purpose is to standardize the reimbursement process for public healthcare services. In brief, DRGs are designed to reflect the relative cost of treating patients with similar conditions. Each DRG is assigned a relative weight or severity level based on the average resources required to treat patients within that group. Hospitals receive reimbursement based on the DRG assigned to each patient. The reimbursement amount is calculated by multiplying the hospital's base payment rate by the relative weight of the assigned DRG. This payment system incentivizes hospitals to efficiently manage resources and provide cost-effective care while ensuring appropriate reimbursement for services rendered.

The fundus photography and OCT economic cost values were collected by assessing the DRG- weights associated with the screening model (Table 1). This also constituted the direct costs in our estimation, though OCT costs did not include more than the consultation costs. Based on national guidelines, from an extended healthcare perspective, we included some indirect costs e.g., two-way transport costs, costs associated with patient time spent at the screening center, and travel time. We collected transport costs and patient-time costs from the Norwegian Medicine Agency (NOMA) database (Legemiddelverk, 2021)^22^. This database serves as a centralized repository of information and regulatory documentation for healthcare professionals, regulatory authorities, and the general public.

**Table 1:** Direct and indirect unit costs for detecting DME in DR screening.

Item	Unit Cost (U.S. dollars)
Fundus photography	158
OCT	153
Transport cost	70
Cost for patient time per hour	23

Conversion rate to U.S. dollars as per January 4, 2024 exchange rate (i.e., 10.40 Norwegian Kroner (NOK), equaling 1 U.S. dollar).

In the sequential screening model, we summated the product of DRG weight and costs for fundus photography and the percentage of patients who presented with diabetic maculopathy and had OCT performed on a scheduled date. For the combined screening model, we added the direct costs of the screening (DRG weighted costs) for all patients. For both screening models, we then added the costs of transportation to and from the screening center, whereas the sequential screening model also included travel costs of the second exam with OCT. We estimated patient time for only fundus photography to be 1.5 h, only OCT to be 1.25 h, and combined fundus photography and OCT to be 1.58 h. We multiplied these estimated times by the patient time cost from the NOMA database and added the product to the total cost.

In a deterministic sensitivity analysis, we evaluated the effect of a range of values on our results. In a one-way sensitivity analysis, we evaluated the impact of a lower or higher sensitivity of fundus photography on the cost per patient with diabetic maculopathy, the total cost of detecting DME, and the overall costs. As regards to the OCT cost, we evaluated the impact of increasing this cost in a two-way sensitivity model and varied the percentage of diabetic maculopathy.

## Results

The study included 90 patients (180 eyes), of whom 12 had T1D and 78 had T2D. Table 2 shows the prevalence of diabetic maculopathy on fundus photography and DME on OCT in the studied population.

**Table 2:** Distribution of the number of patients and eyes when signs of diabetic maculopathy on fundus photography or diabetic macular edema on OCT were present or absent.

		Fundus photography			
		No diabetic maculopathy, N eyes (N patients)	Diabetic maculopathy, N eyes (N patients)	Microaneurysm, N eyes (N patients)	Hard exudate (s), N eyes (N patients)
OCT	No diabetic macular edema, N eyes (N patients)	153 (72)	20 (12)	20 (12)	1 (1)
	Diabetic macular edema, N eyes (N patients)	0	7 (6)	7 (6)	3 (3)

In the T1D group, 9 eyes (37.5%) of 5 patients had diabetic maculopathy on fundus photography. All eyes had MAs within 1 DD from foveola, and one of them additionally displayed hard exudates. Three of these eyes (33.3%) of 2 patients also had DME on OCT. In the T2D group, 18 eyes (11.5%) of 13 patients had diabetic maculopathy on fundus photography. All eyes had MA within 1 DD from foveola, and 3 eyes additionally displayed hard exudates. Four of these eyes (22.2%) of 4 patients also had DME on OCT. Altogether, 7 out of 27 eyes (25.9%) with diabetic maculopathy on fundus photography had DME on OCT. Conversely, no eyes without diabetic maculopathy on fundus photography had DME on OCT.

In the existing sequential DR screening strategy, patients with diabetic maculopathy in one or both eyes seen on fundus photography would require a second appointment for OCT. From a health-economic perspective, 18 patients (20.0%) in such a strategy would have needed a second appointment for OCT, and 6 of these patients (33.3%) would have had DME on OCT.

The cost per patient for sequential fundus photography and OCT was US$ 531, which was higher than the cost per patient for combined fundus photography and OCT (US$ 486), i.e., combining fundus photography and OCT resulted in a cost-saving of US$ 45 per patient; the main cost driver was transportation. If one assumes a one-year screening program of 1250 patients with a 20% prevalence of diabetic maculopathy, the total cost-saving of combining fundus photography and OCT would be US$ 55,654. For patients with diabetic maculopathy on fundus photography, the total cost-saving would be US$ 11,131 per year, whereas the cost-saving for patients with DME on OCT would be US$ 3710 per year.

**Figure 1:**
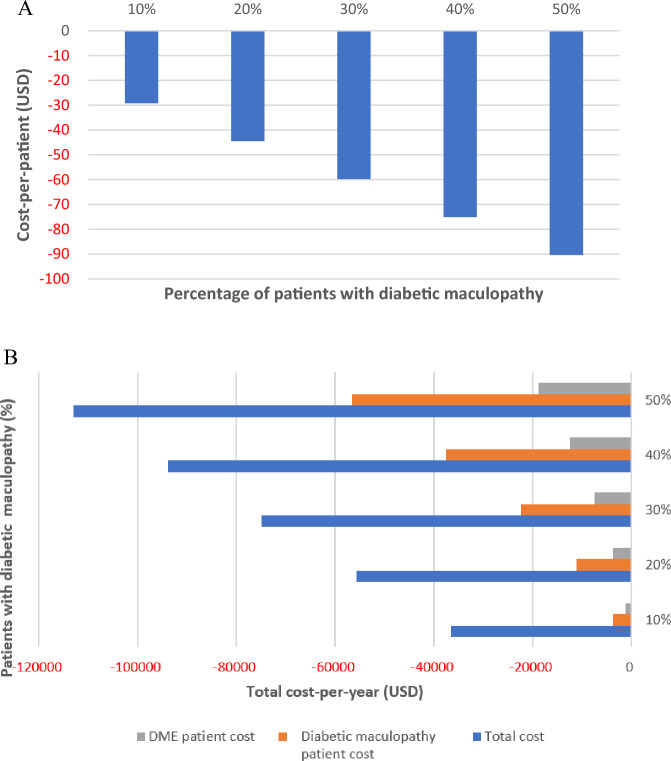
One-way sensitivity analysis for cost difference per patient (**A**) and total costs (**B**) for various percentages (10–50%) of detected diabetic maculopathy. Sequential compared to combined screening.

In a deterministic one-way sensitivity analysis for various percentages (10–50%) of detected diabetic maculopathy, we found that the cost-saving for the combined screening model increased proportionally when tested on the cost-per-patient basis (Fig. 1A); similarly, the total cost per year and the total cost for detected diabetic maculopathy or DME patients per year increased proportionally (Fig. 1B).

**Figure 2:**
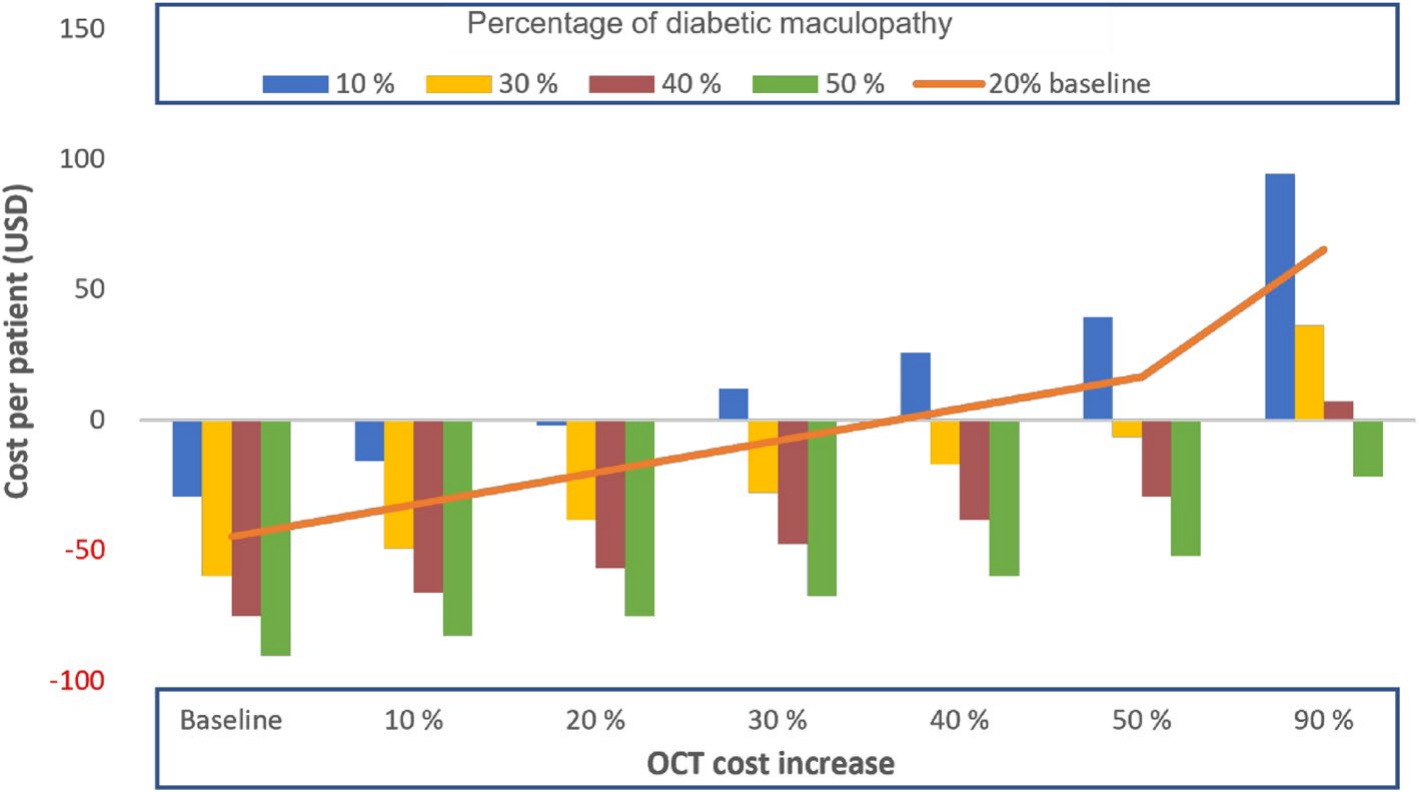
Two-way sensitivity analysis for cost difference per patient by the detected diabetic maculopathy and the OCT cost increase for sequential compared to combined screening.

When assessing the OCT cost in a two-way sensitivity analysis with various percentages of detected diabetic maculopathy (10–50%, with 20% being our study prevalence marked in orange), the cost-per-patient remained cost-saving for the combined screening model until the OCT cost increase was not higher than 35%, and the detected diabetic maculopathy patients remained at 20% (baseline) or more (these values were determined by the crossing of the orange line and the x-axis). Even when the cost of OCT would increase up to 50%, and the percentage of detected diabetic maculopathy would be between 30 and 50%, the combined screening would still remain cost-saving (Fig. 2).

## Discussion

The findings of our study shed light on the importance of utilizing both fundus photography and OCT in DR screening. The results determined the prevalence of diabetic maculopathy and diabetic macular edema (DME) in patients with T1D and T2D enrolled in a pilot DR screening program in the Oslo region, Norway. Only one-fourth of eyes with diabetic maculopathy, or one-third of patients with diabetic maculopathy, had DME on OCT. Compared to the existing DR screening strategy in our department, one-fifth of the patients screened would have needed a new appointment for OCT, of whom one-third would have had DME on OCT requiring referral for treatment evaluation. This highlights the complementary nature of the two imaging modalities in capturing different aspects of diabetic eye disease. In this pilot, we also assessed the cost-effectiveness of routinely combining fundus photography and OCT in DR screening and found this approach to be cost-saving.

Around half of the patients were newly diagnosed with DM (i.e., within 5 years from diagnosis for patients with T1D and within 1 year from diagnosis for patients with T2D), which likely affects the prevalence of diabetic maculopathy and DME in our study. A previous study from the UK showed that less than a quarter of referred M1 cases (defined as exudates within 1 DD of the fovea or MAs/hemorrhages within 1 DD of the fovea, if visual acuity was poorer than 6/12) were found to have clinically significant macular edema (CSMO) in need of treatment^29^. Another study found that around 40% of patients with evidence of diabetic maculopathy on fundus photography had DME on OCT^13^. Moreover, a more recent UK study revealed absence of macular oedema and CSMO on OCT in more than 80% of the patients with M1^30^, whereas a US study suggested that the prevalence of eyes with DME and CSMO detected on fundus photography may be much greater than the prevalence of DME on OCT^14^.

Comparing studies is challenging due to varying definitions of diabetic maculopathy on fundus photography and DME on OCT, as well as population differences. In our study, the presence of only MA within 1 DD of the fovea was also defined as diabetic maculopathy, and we found a prevalence of 20%. In other studies, the prevalence of diabetic maculopathy varies from 10.1 to 61.4%^14,31^.

In the past, stereoscopic fundus photography was utilized to classify DME on the basis of central retinal thickening, either with or without hard exudates. Nowadays, most screening programs employ 2D images. Surrogate markers of DME are then employed, such as hard exudates and retinal hemorrhages/MAs within one DD of the fovea^12–14^. According to the UK National Health Service, diabetic maculopathy (M1) is present in the case of exudates/an area of exudates that is greater than or equal to half the disc area and all within the macular area^32^. Regarding OCT, we defined DME qualitatively as the presence of cysts or subretinal fluid on cross-sectional images. By contrast, other studies have defined DME as intraretinal cyst(s), subretinal fluid, increased central retinal thickness (CRT), or change in internal limiting membrane (ILM) contour^12–14^.

In the absence of diabetic maculopathy on fundus photography, none of the eyes exhibited DME on OCT, emphasizing the specificity of OCT in identifying diabetic macular complications. High specificity is important for optimizing DR screening, thereby relieving the pressure on the health system. Furthermore, the implications of our findings extend beyond clinical considerations to health economic perspectives. Our cost analysis revealed that the combined screening approach with fundus photography and OCT was more cost-efficient than a sequential approach, yielding a cost-saving of US$ 45 per patient. Notably, the main cost driver was transport.

The cost analysis of the combined screening approach remained robust across various scenarios in a deterministic one-way sensitivity analysis. Even with fluctuations in the prevalence of diabetic maculopathy, the combined screening approach consistently demonstrated cost-savings, emphasizing its economic viability. Thus, the implementation of combined screening is likely going to enhance treatment capacity and reduce administrative requirements.

The impact of cost variations of OCT and changes in the prevalence of diabetic maculopathy underscored the resilience of the combined screening approach. As long as the cost increase of OCT did not exceed 35% and the prevalence of diabetic maculopathy was at least 20%, the combined screening approach remained cost-saving. Nevertheless, for instances where the OCT cost would increase by up to 50%, that would require higher prevalence of diabetic maculopathy (e.g. 30–50%) for the combined screening to remain cost-saving. Consequently, we advocate combining fundus photography and OCT in DR screening. It should also be noted that we used separate devices for fundus photography and OCT in this study, but novel integrated devices could further improve the cost efficiency of combining fundus photography and OCT, particularly by reducing the time of each screening visit. The nominal costs of such new devices equal the cost of a fundus camera. An alternative cost-saving approach to always combine fundus photography and OCT would be to train the clinical photographer to evaluate whether diabetic maculopathy is present and only perform OCT in these patients. The new guidelines for DR screening in Norway now recommend this strategy similar to that implemented in Denmark and the UK^21–24^. Our pilot showed that none of the eyes without diabetic maculopathy on fundus photography exhibited DME on OCT. This indicates the safety of performing OCT only on patients with diabetic maculopathy.

The impact of our results reaches beyond the clinical setting to the broader healthcare system. By optimizing the use of imaging technologies such as fundus photography and OCT in DR screening, healthcare providers can improve patient outcomes and reduce the economic burden of diabetic eye complications. Early detection of diabetic maculopathy and DME through a combination of imaging modalities enables targeted interventions that can preserve vision and quality of life for individuals with diabetes.

A limitation of our study is the relatively small number of patients included. Moreover, we did not include visual acuity as an outcome measure since the existing sequential DR screening strategy in our department did not encompass visual acuity testing; the main focus of our study was to compare the two screening strategies.

## Conclusions

Our study of a pilot DR screening program yields valuable insights into the prevalence of diabetic maculopathy and DME in our population. Moreover, the study sheds light on the economic advantages associated with integrating fundus photography and macular OCT into a same-day DR screening protocol. By demonstrating the cost-effectiveness of this combined approach, our findings offer practical guidance for healthcare policymakers and providers seeking to optimize DR screening strategies. By striking a balance between efficiency and cost-effectiveness, these insights might facilitate the development and implementation of more streamlined and sustainable DR screening programs, ultimately enhancing the delivery of quality eye care for individuals with diabetes.

## Data Availability

The authors possess all primary data and agree with allowing the journal review the data upon request to Dr. Ellen Steffenssen Sauesund: ellen.s.sauesund@gmail.com.
